# Strong influence of magnetic field and non-uniform stress on elastic modulus and transition temperatures of twinned Ni–Fe(Co)–Ga alloy

**DOI:** 10.1038/s41598-024-62909-z

**Published:** 2024-05-28

**Authors:** Anna Kosogor, Viktor Soprunyuk, Sabri Koraltan, Vladimir Golub, Dmytro Velyhotskyi, Volodymyr Chernenko, Hideki Hosoda, Dieter Suess, Wilfried Schranz, Victor A. L’vov

**Affiliations:** 1 Faculty of Physics, University of Vienna, Boltzmanngasse 5, 1090 Vienna, Austria; 2 Institute of Magnetism NASU and MESU, Kyiv, 03142 Ukraine; 3 Vienna Doctoral School in Physics, University of Vienna, Boltzmanngasse 5, 1090 Vienna, Austria; 4 Research Platform MMM Mathematics-Magnetism-Materials, University of Vienna, Vienna, Austria; 5 BCMaterials, Basque Center for Materials, Applications and Nanostructures, University of Basque Country, UPV/EHU Science Park, 48940 Leioa, Spain; 6 Institute of Innovative Research (IIR), Tokyo Institute of Technology, Yokohama, 226-8503 Japan; 7 Ikerbasque, Basque Foundation for Science, 48009 Bilbao, Spain

**Keywords:** Martensitic transformation, Martensite reorientation, Magnetization, Electric resistivity, Landau-type theory, Magnetic properties and materials, Phase transitions and critical phenomena

## Abstract

The magnetization value and electric resistivity of the single-crystalline sample of Ni_50_Fe_19_Co_4_Ga_27_ shape memory alloy were measured. The elastic modulus was determined by the Dynamic Mechanical Analysis (DMA). The characteristic temperatures of martensitic transformation (MT) of the alloy were estimated from the temperature dependences of magnetization, electric resistivity and elastic modulus. A significant disparity between MT temperatures resulting from DMA and those estimated from magnetic and resistivity measurements was discovered. It was argued that the discrepancy is caused by the non-uniform mechanical stressing of twinned single crystal by the DMA analyzer. Moreover, the DMA measurements revealed a significant decrease of the elastic modulus of twinned martensite under the applied magnetic field of 1.5 kOe. To explain this effect, the temperature-dependent Young’s modulus of twinned crystal lattice was computed. The computations showed that the experimentally observed field-induced change of the elastic modulus is caused by the stress-assisted detwinning of the crystal lattice by the applied magnetic field.

## Introduction

Ferromagnetic shape memory alloys (SMAs) undergo the first-order martensitic transformations (MTs) from a high-temperature cubic austenitic phase to a low-temperature martensitic phase with reduced symmetry in response to the changes of temperature, mechanical stress and/or magnetic field. SMAs have been widely used in various applications, such as biomedical devices, actuators, and sensors^1–3^. Intensive studies of ferromagnetic SMAs began when the large (~ 6%) magnetic-field-induced strain (MFIS) was discovered in the martensitic phases of Ni–Mn–Ga single crystals under moderate (≤ 5 kOe) magnetic fields^4–6^. The observed value of MFIS was increased then up to 12% due to the purposeful research (see Ref.^7^ and references therein). The MFIS is caused by the detwinning of the twinned single crystal of SMA due to the field-induced motion of twin boundaries. This process is known as the martensite reorientation^4,8^. The field-induced martensite reorientation is described theoretically in terms of the magnetic-field-induced stress (magnetostress)^9^ caused by magnetoelastic interaction^10^. The magnetostress acts on the twinned martensite similarly to the mechanical stress induced by axial mechanical load. Thus, the magnetostress triggering the magnetically induced twin reorientation corresponds to the mechanical stress value starting the martensite reorientation during stress–strain tests. The observation of maximum value of MFIS became possible due to the remarkably high mobility of twin boundaries and the exceptionally low twinning stress inherent to Ni–Mn–Ga alloys^4,11,12^. Nevertheless, due to the high brittleness of Ni–Mn–Ga alloys, investigations were prompted into alternative systems, such as Ni–Fe–Ga exhibiting better mechanical properties.

Ni–Fe(Co)–Ga magnetic shape memory alloys attract the attention of researchers due to their unusual anhysteretic stress‒strain behavior: the huge (up to 14%) reversible strain and hysteresis of about 1 MPa was observed in the course of stress‒strain cycle^13,14^. The narrow hysteresis was explained using the Landau-type theory of martensitic phase transitions, which showed the narrowing of the interval of stresses, corresponding to the mixed austenitic-martensitic state on approach to the critical point in the stress-temperature phase diagram^13,15^. The Ni–Fe(Co)–Ga alloys demonstrate the prominent properties such as two-way shape memory effect^16^, excellent superelastic behavior on macro- and microscale, magnetoresistance^17^ and giant elastocaloric effect^18^. The Ni–Fe(Co)–Ga single crystals appeared to be less brittle than Ni–Mn–Ga, which makes them promising for applications.

The unstressed Ni–Fe(Co)–Ga alloy does not exhibit the magnetic-field-induced reorientation of martensitic variants because the threshold value of mechanical stress required to initiate martensite reorientation exceeds the achievable value of magnetostress. However, a strain value of 8.5% was observed in the Ni_49_Fe_18_Co_6_Ga_27_ alloy due to the cooperative effect of a magnetic field of 4 kOe and a compressive stress of 8 MPa^19^.

The aforementioned properties underscore the growing interest in investigating the functional properties of Ni–Fe(Co)–Ga alloys and other ferromagnetic SMAs under the combined influence of various external factors, including magnetic fields, mechanical loading, hydrostatic pressure etc.

In the present study, the combined influence of magnetic field and non-uniform stress on transformational and elastic properties of Ni–Fe(Co)–Ga single-crystalline sample are investigated. The results of magnetic and resistivity measurements are compared with the Dynamic Mechanical Analysis (DMA) data. A theoretical analysis based on the Landau-type theory showed that the field-induced decrease of elastic modulus observed by DMA technique can be attributed to the influence of magnetic field on the twin structure of the martensitic phase.

## Experimental

The single crystal ingot was grown with an optical floating zone furnace using an ingot of the Ni_50_Fe_19_Co_4_Ga_27_ (at.%) alloy fabricated by the arc melting. The sample of rectangular geometry 12.5 × 1.5 × 0.3 mm^3^ with all {001} faces had been prepared by a spark-cut erosion from single-crystalline ingot and then heat treated at 1373 K for 24 h with a subsequent quenching into water. The phase transformation from cubic ferromagnetic phase (austenite) to tetragonal ferromagnetic phase (martensite) was induced by cooling of the sample. The X-ray diffraction pattern was measured at 140 K. The L1_0_ crystal structure with lattice parameters $c_{{{\text{L}}1_{0} }} \approx 0.326\,{\text{nm}}$ and $a_{{{\text{L}}1_{0} }} \approx 0.380\,{\text{nm}}$ ($c \approx 0.652{\text{ nm}}$ and $a \approx 0.536{\text{ nm}}$ in terms of a body-centered tetragonal unit cell) was confirmed in a good agreement with the values reported previously for the similar alloys^20,21^.

Magnetic measurements were carried out in the temperature range 100–400 K with a heating/cooling rate of 2 K/min, using a physical properties measurement system (PPMS), Quantum Design Ltd. The resistivity measurements were performed using standard four-probe method on home-built experimental setup at constant current 1 mA during the heating up and cooling down with the rate 2 K/min.

**Figure 1:**
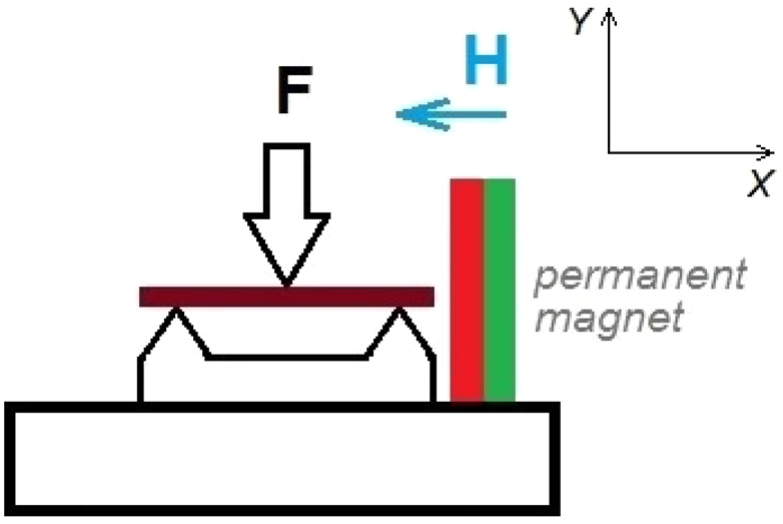
Sketch of the measurement geometry (three-point bending) and direction of the magnetic field **H** applied along the [100] crystallographic axis of the sample, which is parallel to its longest edge.

The elastic properties of the Ni_50_Fe_19_Co_4_Ga_27_ sample were studied using a dynamic mechanical analyzer (Diamond DMA—Perkin Elmer Inc.) in three-point bending geometry (Fig. 1). The sample was exposed to a static force, which was modulated by a variable force of fixed amplitude of 1 N and frequency *f* of 1 Hz. The strain amplitude *u* of the forced elastic vibrations of the sample and the phase shift $\delta$ between the amplitude and force were registered via inductive coupling with a resolution of $\Delta u = 10{\text{ nm}}$ and $\Delta \delta \approx 0.1$°.

The experiments were performed in the temperature range from 130 to 460 K with cooling and heating rate of 2 K/min at zero magnetic field and under the stationary magnetic field applied parallel to the longest edge of the sample (Fig. 1). The magnitude of the magnetic field at the sample’s location was measured by a Voltcraft Magnetic field analyzer GM-70. Several cooling/heating cycles, both in the absence of a magnetic field and in the magnetic field, confirmed the good reproducibility of the DMA dependences.

## Results

### Magnetic measurements

**Figure 2:**
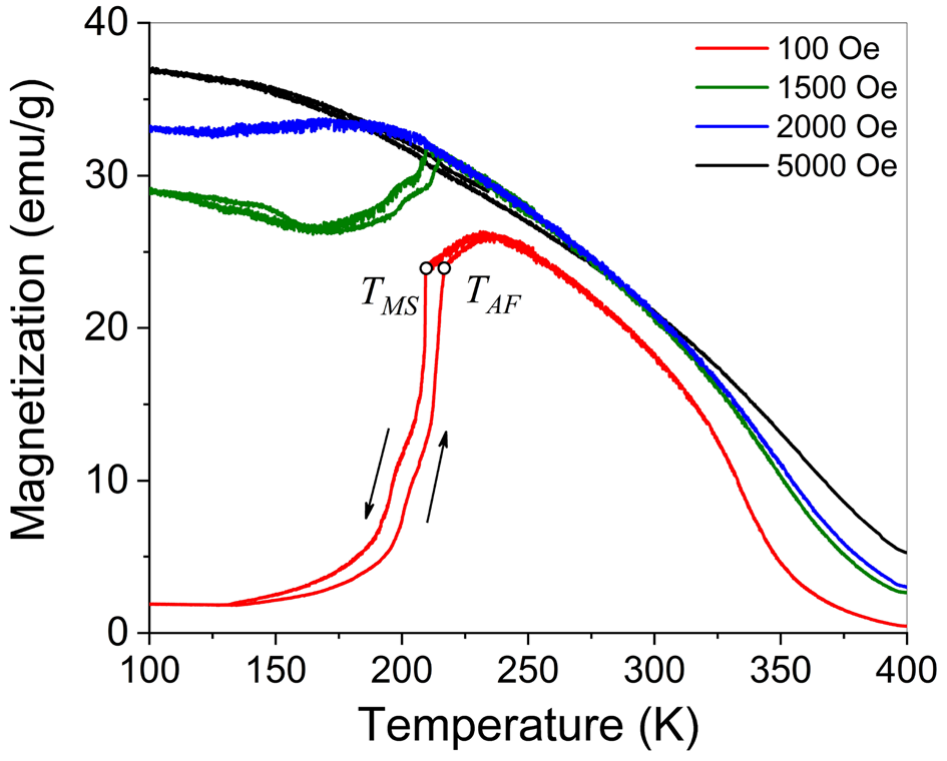
Temperature dependences of magnetization measured for Ni_50_Fe_19_Co_4_Ga_27_ single crystal in different magnetic fields applied in the [100] direction (parallel to the longest edge of the sample).

Figure 2 shows the temperature dependences of magnetization measured during cooling and heating of the Ni_50_Fe_19_Co_4_Ga_27_ alloy under various magnetic fields directed along the sample's longest edge, parallel to the [100] crystallographic direction of the cubic phase. Martensitic transformation (MT) from the cubic austenitic phase to the tetragonal martensitic phase results in the decrease of the magnetization. Such magnetization behavior was already reported for Ni_2_MnGa in an early publication^22^. It was shown that the decrease of magnetization at MT in a non-saturating magnetic field is caused by the appearance of uniaxial magnetocrystalline anisotropy in the tetragonal phase of ferromagnetic SMA and enables an estimation of the magnetocrystalline anisotropy and magnetoelastic constants of the alloy^23^. In the field of 100 Oe, the sharp decrease/increase of magnetization value starts/finishes at the temperatures of 210 K/216 K on cooling/heating of the specimen, respectively (see Fig. 2). These MT temperatures correspond to martensite start $T_{MS}$ and austenite finish $T_{AF}$ temperatures, respectively.

### Resistivity measurements

**Figure 3:**
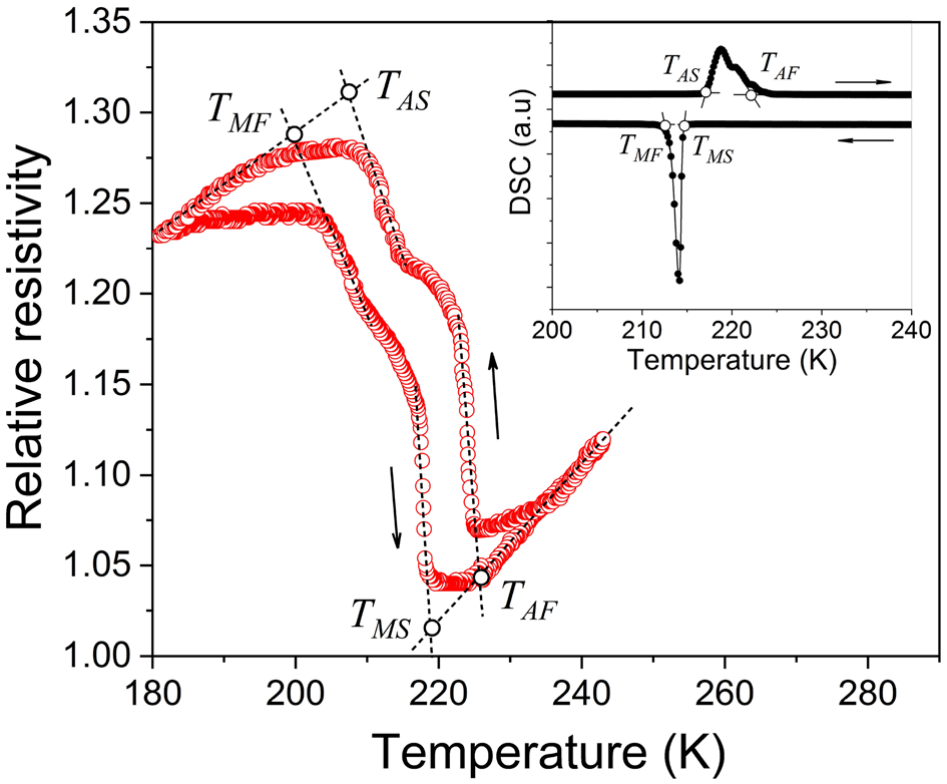
Relative values of electrical resistance vs. temperature measured for Ni_50_Fe_19_Co_4_Ga_27_ alloy specimen. The points corresponding to characteristic temperature values are shown by circles. Inset: calorimetry data obtained in Ref.^13^ for another sample of Ni_50_Fe_19_Co_4_Ga_27_ alloy.

The experimental temperature dependence of electrical resistance is commonly used for the determination of MT temperatures (see e.g. Ref.^24^). Figure 3 shows the temperature dependence of relative value of electrical resistance measured for the Ni_50_Fe_19_Co_4_Ga_27_ alloy specimen. The temperatures $T_{MS} = 218{\text{ K}}$ and $T_{MF} = 204{\text{ K}}$ of start and finish of the forward (martensite to austenite) transformation and the temperatures $T_{AS} = 208{\text{ K}}$ and $T_{AF} = 225{\text{ K}}$ of start and finish of the reverse (austenite to martensite) transformation were determined as it is shown in Fig. 3. The inset in Fig. 3 displays the differential scanning calorimetry (DSC) data obtained from Ref.^13^ for another sample of Ni_50_Fe_19_Co_4_Ga_27_ alloy. The MT temperatures determined from magnetic, resistivity and calorimetry data are listed in Table 1.

**Table 1:** Characteristic temperatures of the forward and reverse MTs estimated from magnetic and resistivity measurements, DSC and DMA data.

	*T*_*MS*_, K	*T*_*MF*_, K	*T*_*AS*_, K	*T*_*AF*_, K
DMA	188	170	190	205
Magnetic	210	–	–	216
Resistivity	218	201	208	225
DSC^a^	214	212	217	222

^a^Values measured in Ref.^13^ for another sample of the Ni_50_Fe_19_Co_4_Ga_27_ alloy.

### Dynamic mechanical analysis

**Figure 4:**
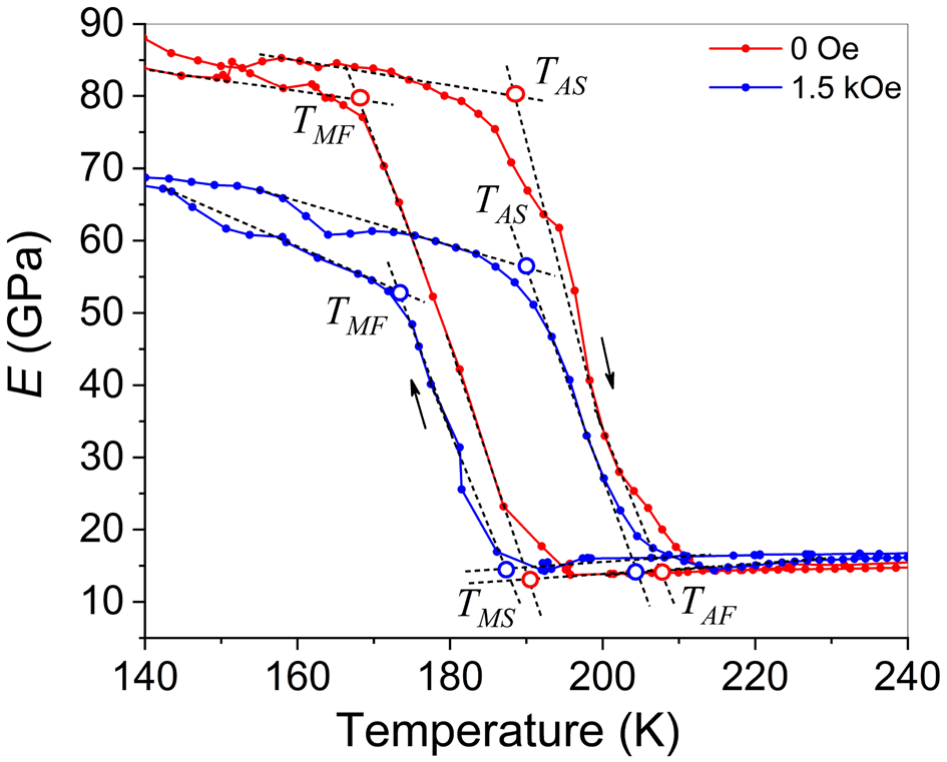
Temperature dependences of Young’s modulus obtained in zero magnetic field (red curve) and in the magnetic field of 1.5 kOe (blue curve).

The temperature dependences of Young’s modulus, *E*, of Ni_50_Fe_19_Co_4_Ga_27_ alloy were measured in zero magnetic field and in the field of 1.5 kOe, induced by a permanent magnet. The temperature behavior of *E* during both cooling and heating cycles is illustrated in Fig. 4.

The curves in Fig. 4 enabled the determination of temperatures of start and finish of forward MT and reverse MT by the two-tangent method as shown in the figure. These temperatures $T_{MS} = 188 \pm 2{\text{ K}}$, $T_{MF} = 170 \pm 2{\text{ K}}$, $T_{AS} = 190{\text{ K}}$ and $T_{AF} = 205 \pm 2{\text{ K}}$ are also presented for comparison with the MT temperatures determined from different experiments in Table 1. The characteristic MT temperatures obtained in zero magnetic field and in the field of 1.5 kOe are almost the same within the experimental error. It should be noted that while there is some ambiguity in the precise determination of MT temperatures, Table 1 illustrates that the MT temperatures obtained from DMA are notably lower than those estimated from magnetic, resistivity and calorimetry measurements. Specifically, the table indicates that the maximum discrepancy between magnetic, resistivity, and DSC measurements is 8 K, 11 K, 9 K, and 9 K for $T_{MS}$, $T_{MF}$, $T_{AS}$ and $T_{AF}$ temperatures (respectively), whereas the maximum deviation of these measurements from DMA data is 30 K, 42 K, 27 K, and 20 K (respectively). The discussion addressing this discrepancy in the values obtained from DMA and other measurements will follow below.

As can be seen in Fig. 4, the DMA results indicate a significant decrease in the elastic modulus of the martensite due to the magnetic field action. It should be noted that resonant ultrasound spectroscopy was used to explore the influence of a magnetic field on the shear elastic modulus $C^{\prime}$, which is related to Young’s modulus as $C^{\prime}(T) \approx E(T)/3$, in the austenitic phase of Ni–Mn–Ga alloy^25,26^. However, to the best of our knowledge, the temperature dependence of the low-frequency Young's modulus of martensite in ferromagnetic SMAs in magnetic field has not been investigated previously. The following section will elucidate the origin of the observed strong decrease in the elastic modulus of the martensitic phase.

## Theoretical explanation of experimental results

**Figure 5:**
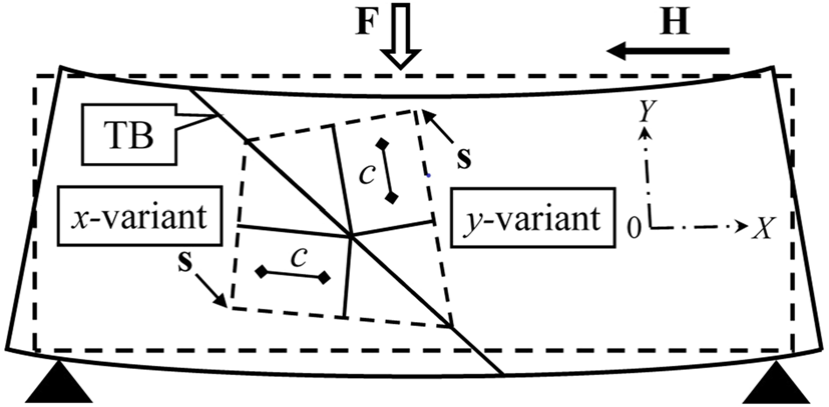
Schematic presentation of the cross-section of the twinned sample depicted in Fig. 1: TB is twin boundary, the **s-**vectors indicate the directions of shear of the atomic planes, which transform the cubic crystal lattice into the *x*- and *y*-variants. The two-side arrows show the directions of the four-fold symmetry axes of the *x*- and *y*-variants of crystal lattice with *a* < *c*.

The edges of the sample depicted in Fig. 1 are oriented in $\left\langle {100} \right\rangle$ crystallographic directions. Let the coordinate axis *OX*||[100] be parallel to the longest edge of the sample, the coordinate axis is *Y* perpendicular to the 12.5 × 1.5 mm^2^ face, and let $y = 0$ corresponds to the middle of the sample plane. The appearance of the twin structure formed by the alternating spatial domains of the tetragonal lattice with *c*||*OX* and *c*||*OY* is preferable because this structure arises from the cubic lattice by periodic shearing of atomic planes in the opposite directions parallel/antiparallel to $[\overline{1}10]$ and $[1\overline{1}0]$ crystallographic direction, i.e., at 45° to the face of the sample (see Fig. 5).

The lattice parameters satisfy the condition $a < c$ in the martensitic phase of the Ni_50_Fe_19_Co_4_Ga_27_ alloy. The DMA analyzer applies a variable non-uniform mechanical stress $\sigma$ (with a maximum value of $\sigma_{\max } \approx 30{\text{ MPa}}$) and bending strain $\varepsilon_{ik} (y)$. The strain component $\varepsilon_{xx} (y)$ is positive at $y < 0$ and negative at $y > 0$, because the lower face of the sample is elongated and upper face is shortened in *x-*axis direction. Therefore, the bending strain is advantageous for the formation of the *x*-variant of the martensitic phase at $y < 0$ and for the *y-*variant at $y > 0$. However, the tendency to formation of the *x-*variant of the tetragonal lattice in the lower layer of the *thin plate* and the *y-*variant in its higher layer is *disadvantageous in the kinetic aspect*. It can be assumed, therefore, that the *xy-*twins, piercing through the plate, appear if the thin plate is overcooled below the MT temperature measured in the unstrained plate. This scenario offers a probable explanation for the difference in MT temperatures estimated from DMA and magnetic/resistivity measurements.

To describe the magnetic field influence of temperature dependence of Young’s modulus, one can take into account, that the longitudinal magnetostriction constant of Ni‒Fe‒Ga alloys is negative^27^. It should be expected, therefore, that the magnetic field **H**||*OX* promotes the formation of spatial domains of the tetragonal lattice with *c*||*OY*, *a*||*OX* because the forced magnetostriction results in the contraction of the alloy sample in *OX* direction. Due to this, the magnetic field application to the twinned tetragonal lattice should decrease the volume fraction $\alpha$ of the twin component with *c*||*OY* (*y*-variant of martensitic phase) by the value $\Delta \alpha (H)$. The change of $\alpha$ under mechanical stress or/and magnetic field is referred to as martensite reorientation.

As it was mentioned before, the field-induced martensite reorientation is described theoretically in terms of the magnetostress thermodynamically conjugated to magnetostrictive strain through the shear elastic modulus of the alloy^6,9,10^. The equivalent magnetostress, $\sigma_{{{\text{eq}}}} (H)$, has a similar influence on the twin structure as the mechanical stress, $\sigma$, induced in the twinned sample of the alloy by the axial mechanical load. It is important to note, that the martensite reorientation starts at a “threshold” value of mechanical stress, $\sigma_{th}$, or magnetic field, $H_{th}$. For Ni–Mn–Ga SMAs, which exhibit the almost complete martensite reorientation under magnetic field, $\sigma_{th} \sim 2{\text{ MPa}}$, $H_{th} \sim 3{\text{ kOe}}$^6^, $\sigma_{{{\text{eq}}}} (H_{S} ) \approx 3{\text{ MPa}}$, where $H_{S}$ is a magnetic saturation field^28^, while for the majority of ferromagnetic SMAs the field-induced martensite reorientation is not observed, i.e., $\sigma_{{{\text{eq}}}} (H_{S} ) < \sigma_{th}$. However, in the course of the present DMA measurements carried out in magnetic field, the alloy is stressed by the total stress, being the sum of variable mechanical stress and magnetostress. If the *total* stress exceeds the threshold value, the martensite reorientation can start even if $\sigma_{{{\text{eq}}}} (H_{S} ) < \sigma_{th}$. In this case the total mechanical stress changes the twin structure arising below MT temperature. As previously stated, the Ni_49_Fe_18_Co_6_Ga_27_ alloy achieved a significant MFIS of 8.5% under a magnetic field of 4 kOe, with assistance from an external compressive stress of 8 MPa^19^. Subsequently, a MFIS of 2% under a magnetic field of 15 kOe was demonstrated for the same alloy, this time with a tensile stress of 16 MPa applied to the alloy sample^29^. However, in its unstressed state, the alloy does not demonstrate the reorientation of martensite variants induced by a magnetic field and associated with it MFIS. These experiments lend support to our assumption regarding martensite reorientation/detwinning in the Ni_50_Fe_19_Co_4_Ga_27_ alloy under the combined influence of magnetic field and mechanical stress induced by DMA. The maximum stress induced by DMA is approximately 30 MPa, closely aligning with experimental conditions^19,29^. This observation suggests the potential for observing MFIS when alternating stress is applied to the alloy sample.

Figure 4 shows a noticeable influence of magnetic field on the Young’s modulus of the martensitic phase in the Ni_50_Fe_19_Co_4_Ga_27_ alloy. The Landau-type theory has previously been employed to quantitatively model the temperature dependence of Young’s modulus measured during DMA tests, showing good agreement between experimental and theoretical results^30^. The mathematical expressions for the elastic modules of the martensitic structure formed by two alternating variants of tetragonal phase were obtained in Ref.^31^ using the following Landau expansion for the Helmholtz free energy

1 $$\begin{aligned} F & = \frac{1}{2}c_{2} (T)(u_{2}^{2} + u_{3}^{2} ) + \frac{1}{3}a_{4} u_{3} (u_{3}^{2} - 3u_{2}^{2} ) \\ & \quad + \frac{1}{4}b_{4} (u_{2}^{2} + u_{3}^{2} )^{2} , \\ \end{aligned}$$

where $u_{2} = \sqrt 3 (\varepsilon_{xx} - \varepsilon_{yy} )$ and $u_{3} = 2\varepsilon_{zz} - \varepsilon_{yy} - \varepsilon_{xx}$ are the components of the order parameter of the cubic-tetragonal MT, composed from the strain tensor components $\varepsilon_{ik}$. The coefficients $c_{2} ,a_{4} ,b_{4}$ are linear combinations of the second-, third-, and fourth-order elastic modules. The temperature-dependent coefficient $c_{2} (T)$ satisfies the equation

2 $$c_{2} (T) = \left\{ \begin{gathered} E_{A} (T)/9\quad {\text{ if }}T > T_{MS} , \hfill \\ - a_{4} u_{0} (T) - b_{4} u_{0}^{2} (T),\quad {\text{otherwise}}, \hfill \\ \end{gathered} \right.$$

where $E_{A} (T)$ is the temperature dependent Young’s modulus in the *austenitic* phase, $u_{2} = 0$, $u_{3} \equiv u_{0} (T) = 2[c(T)/a(T) - 1]$ are the equilibrium values of the order parameter components expressed through the experimental values of the lattice parameters of the tetragonal phase^32^. The coefficients $a_{4}$, $b_{4}$ can be estimated from the experimental values of Young’s modulus and lattice constants of *martensitic* phase using the equations

3 $$a_{4} = - 2\frac{{E_{M} (T_{MS} )}}{{9[c(T_{MF} )/a(T_{MF} ) - 1]}},b_{4} = \frac{{E_{M} (T_{MS} )}}{{9[c(T_{MF} )/a(T_{MF} ) - 1]^{2} }},$$

where *c*, *a* are the lattice constants measured after the finish of forward MT, $E_{M} (T_{MS} )$ is the Young’s modulus value at the MT start temperature $T_{MS}$^32^. The values $a_{4} = - 20.4{\text{ GPa}}$ and $b_{4} = 47.2{\text{ GPa}}$ result from the experimental lattice parameters ratio $c/a \approx 1.22$ and experimental value of Young’s modulus $E_{M} (T_{MS} ) = 19.9{\text{ GPa}}$ (Fig. 4).

The Young's modulus of the martensitic structure formed by the alternating *x*- and *y*-domains of the tetragonal lattice is expressed as

4 $$E_{M} (T) = 3[3c_{2} (T) + 6\alpha a_{4} u_{0} + 3(\alpha + 2)b_{4} u_{0}^{2} ]g(c_{2} ,u_{0} ,\alpha ),$$

where

$$g(c_{2} ,u_{0} ,\alpha ) = \frac{{4\eta_{1} \eta_{2} }}{{4\eta_{1} \eta_{2} + 3\alpha (1 - \alpha )(\eta_{1} - \eta_{2} )^{2} }},$$

5 $$\eta_{1} = c_{2} + 2a_{4} u_{0} + 3b_{4} u_{0}^{2} ,$$

$$\eta_{2} = c_{2} - 2a_{4} u_{0} + b_{4} u_{0}^{2} ,$$

see Ref.^31^ for more details. The value $\alpha = 1/2$ corresponds to the twin structure with equal volume fractions of *x*- and *y*-domains. The value $\alpha = 1/3$ corresponds to the perfect compatibility of twins, because in the case of shear deformation of cubic crystal lattice $c - a_{0} \approx 2(a_{0} - a)$, where $a_{0}$ is the lattice parameter of the undeformed cubic lattice^31^.

**Figure 6:**
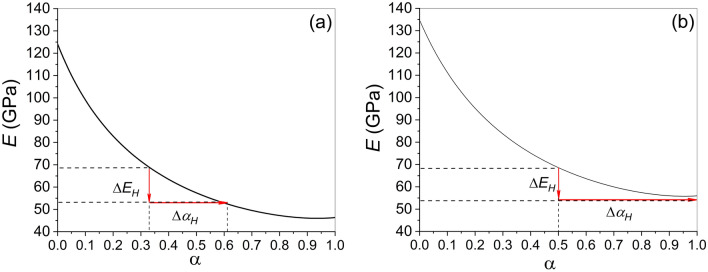
Dependence of elastic modulus *E* on the volume fraction of the favorable martensite variant computed for the value $T = 172{\text{ K}}$ resulting from DMA (see star marker in Fig. 7), and the values $\alpha_{0} = 1/3$ and $\alpha_{0} = 1/2$ (panels (**a**) and (**b**), respectively). Dashed lines correspond to experimentally observed change of elastic modulus in zero magnetic field and at 1.5 kOe.

**Figure 7:**
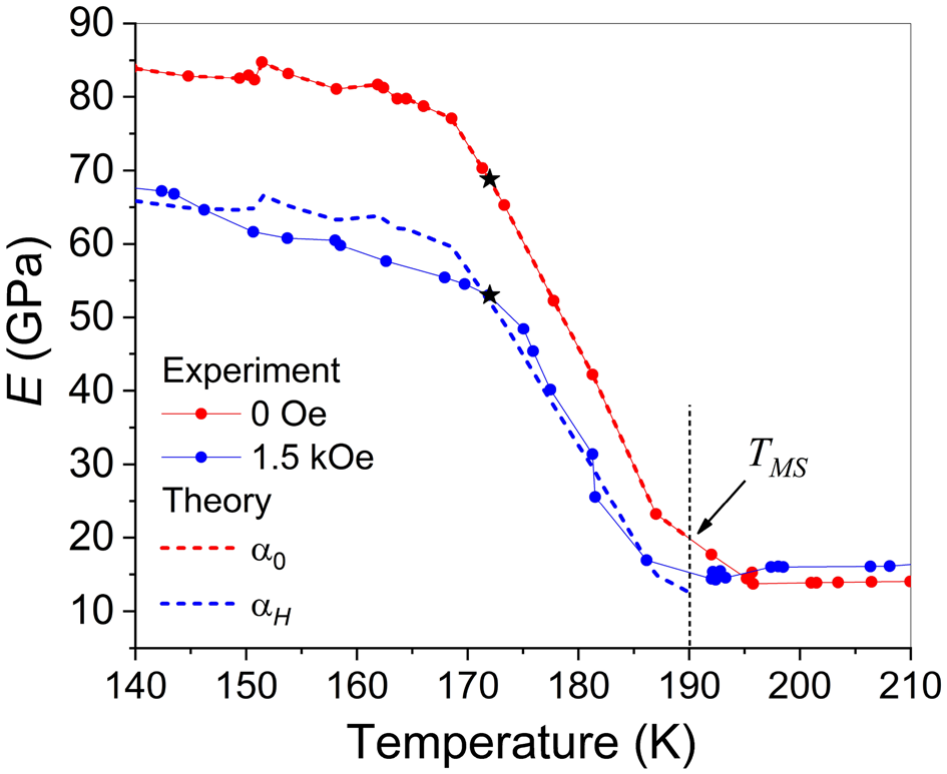
Temperature dependences of Young’s modulus of twinned Ni_50_Fe_19_Co_4_Ga_27_ crystal computed for different volume fractions of twin variants in comparison with experimental dependence of Young’s modulus measured during cooling in zero magnetic field and magnetic field of 1.5 kOe. The vertical dashed line marks the *T*_*MS*_ temperature as a start of MT from cubic austenite to the twinned martensitic structure.

Substituting the $g(c_{2} ,u_{0} ,\alpha )$ function into Eq. (4) one can see that the Eqs. (2), (4) form the equation system for two unknown temperature dependent values $u_{0} (T)$, $c_{2} (T)$. This equation system involves the volume fraction of *y*-variant of martensitic phase $\alpha$, the temperature dependent elastic modulus $E_{M} (T)$ and estimated above coefficients $a_{4}$, $b_{4}$. For theoretical modeling of experimentally observed influence of the magnetic field on the elastic modulus of alloy the experimental temperature dependence of Young's modulus *measured in zero magnetic field* was substituted into Eq. (4) and the values $u_{0} (T)$, $c_{2} (T)$ were computed numerically for $\alpha_{0} = 1/3$, and $\alpha_{0} = 1/2$, considered the probable values of the volume fraction of *y*-variant of martensitic phase *in zero magnetic field*. The dependence of the elastic modulus of twinned crystal on the volume fraction $\alpha$ was computed then from Eqs. (4), (5). The result of computations is shown in Fig. 6. For the sake of specificity, a temperature value $T = 172{\text{ K}}$ was chosen to assess the influence of a magnetic field on the twin structure of martensite (refer to the star marker in Fig. 7). The vertical arrow in Fig. 6 shows the experimentally observed change of the Young's modulus $\Delta E$ under magnetic field of 1.5 kOe. The horizontal arrow illustrates that the magnetic field application results in the increase of volume fraction of favorable variant of martensitic phase from $\alpha_{0} = 1/3$ to $\alpha_{H} = 0.55$ or from $\alpha_{0} = 1/2$ to single-variant state, $\alpha_{H} = 1$.

Figure 7 depicts the experimental temperature dependences of Young's modulus for Ni_50_Fe_19_Co_4_Ga_27_ crystal measured during cooling in zero magnetic field and under a magnetic field of 1.5 kOe together with theoretical temperature dependences of elastic modulus computed from Eqs. (4), (5) for different volume fractions of twin variants ($\alpha = \alpha_{0}$ and $\alpha = \alpha_{H}$). The excellent fit of the theoretical curve to experimental points achieved for $\alpha = \alpha_{0}$ illustrates only the high accuracy of the previously computed $a_{4}$, $b_{4}$ and $u_{0} (T)$ values. The vertical dashed line indicates $T_{MS}$ temperature corresponding to MT start from austenitic phase to the twinned martensitic structure. As seen in Fig. 7, the increase of volume fraction of favorable variant of martensitic phase from $\alpha = \alpha_{0}$ to $\alpha = \alpha_{H}$ leads to the decrease of the elastic modulus of martensitic phase. As so, it can be concluded that the experimentally observed decrease of elastic modulus in magnetic field is caused by the magnetically induced detwinning of the martensitic structure. The reasonable agreement between theoretical and experimental results is demonstrated.

## Discussion

The experiments described above reveal two distinct effects observed for the Ni_50_Fe_19_Co_4_Ga_27_ ferromagnetic shape memory alloy:

(i) a discrepancy between MT temperatures estimated from magnetic, resistivity and calorimetry curves compared to DMA measurements (see Table 1);
(ii) a strong influence of an external magnetic field on the elastic modulus of the martensitic phase.

The feature (i) is observed because the DMA analyzer applies the non-uniform mechanical stress to the twinned alloy specimen and induces the bending deformation (see Fig. 5). The bending deformation is favorable for the increase of the volume fraction of *x*-variant of the tetragonal lattice in the expanded layer of the experimental specimen and for the increase of volume fraction of *y*-variant in its contracted layer. It creates a disadvantage for the kinetics of the phase transition from austenitic phase to the twinned martensitic state of alloy. Consequently, *xy*-twins appear only when the thin plate subjected to the non-uniform stress is overcooled below the MT temperature measured for the unstressed plate. This leads to the differences of MT temperatures obtained using the DMA from the those estimated from the magnetic and resistivity measurements.

Theoretical analysis strongly supports the assumption that feature (ii) is caused by *cooperative influence of the external magnetic field and mechanical stress* induced by DMA technique *on the twin structure of Ni*_*50*_*Fe*_*19*_*Co*_*4*_*Ga*_*27*_ alloy. To support this idea the temperature dependence of the Young’s modulus was computed using the theoretical model proposed in Ref.^31^. The computations showed that even the partial detwinning of the twinned martensitic alloy can provide the observed change of elastic modulus.

The conclusion about the noticeable cooperative influence of the external magnetic field and mechanical stress on the twin structure of Ni_49_Fe_18_Co_6_Ga_27_ alloy, observed in the course of DMA experiments, complements the well-known experimental data showing the variability of the twin structure of ferromagnetic SMAs under magnetic field and *uniform* mechanical stress. In particular, it was shown that a compressive force applied parallel to the magnetic field vector assists the magnetic-field-induced process of martensite reorientation in Ni_49_Fe_18_Co_6_Ga_27_ alloy^19^, while the compressive force applied perpendicular to the magnetic field vector opposes this process in Ni_47.4_Mn_32.1_Ga_20.5_ alloy^6^. The DMA analyzer creates situation which, to the best of our knowledge, was not analyzed yet: the applied force contracts upper layer of the thin platelet of SMA, expands its lower layer and due to this induces the *non-uniform* mechanical stress (see Fig. 5); the magnetic field applied perpendicular to the force vector opposes the formation of *x-*variant of martensitic phase in the contracted layer and *simultaneously* opposes the formation of *y-*variant in expanded layer, because $c > a$. Therefore, the cooperative influence of the magnetic field and *non-uniform* mechanical stress retards the appearance of martensite and lowers the MT temperature.

The reported in the literature variability of the twin structure of ferromagnetic SMAs supports the given above explanation of the discrepancy between the characteristic MT temperatures resulting from DMA and DSC. It should be emphasized, however, that this discrepancy cannot be considered as the error: the DSC gives the *real* temperature values corresponding to the start and finish of quasi-equilibrium phase transformation process, while the DMA gives the *real* temperature values corresponding to the start and finish of non-equilibrium phase transformation in the non-uniformly strained sample.

## Conclusion

It should be concluded that the detwinning process originated by the cooperative influence of magnetic field and mechanical stress on the twin structure of ferromagnetic martensite can not only induce a well-studied giant deformation of ferromagnetic martensite, but can noticeably change the transformational behavior and elastic modulus of ferromagnetic alloy. Due to this, the DMA measurements revealed a significant decrease in the elastic modulus of the martensitic phase under the applied magnetic field of 1.5 kOe. Moreover, mechanical stress induced by DMA technique results in the discrepancy in MT temperatures obtained by DMA and those resulting from the magnetic, resistivity and calorimetry measurements.

## Data Availability

The datasets used and/or analysed during the current study available from the corresponding author on reasonable request.
